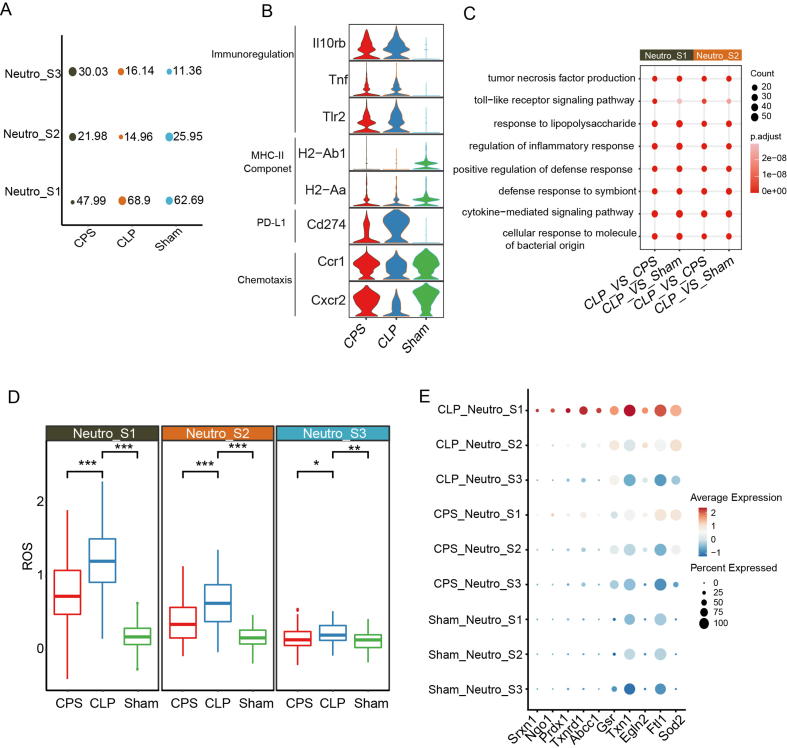# Single-cell transcriptomics illustrates the immune inflammatory responses of septic mice spleen after capsaicin treatment

**DOI:** 10.1016/j.gendis.2024.101256

**Published:** 2024-03-05

**Authors:** Weijin Qiu, Qian Zhang, Jing Liu, Xueling He, Guangqing Cheng, Jiayun Chen, Yunmeng Bai, Piao Luo, Ping Gong, Fei Shi, Jigang Wang

**Affiliations:** Department of Emergency, Shenzhen People's Hospital, The First Affiliated Hospital, Southern University of Science and Technology, Shenzhen, Guangdong 518020, China; Department of Emergency, Shenzhen People's Hospital, The First Affiliated Hospital, Southern University of Science and Technology, Shenzhen, Guangdong 518020, China; School of Traditional Chinese Medicine and School of Pharmaceutical Sciences, Southern Medical University, Guangzhou, Guangdong 510515, China; Department of Emergency, Shenzhen People's Hospital, The First Affiliated Hospital, Southern University of Science and Technology, Shenzhen, Guangdong 518020, China; Department of Emergency, Shenzhen People's Hospital, The First Affiliated Hospital, Southern University of Science and Technology, Shenzhen, Guangdong 518020, China; State Key Laboratory for Quality Ensurance and Sustainable Use of Dao-di Herbs, Artemisinin Research Center, and Institute of Chinese Materia Medica, China Academy of Chinese Medical Sciences, Beijing 100700, China; Department of Infectious Disease, Shenzhen People's Hospital, The First Affiliated Hospital, Southern University of Science and Technology, Shenzhen, Guangdong 518020, China; School of Traditional Chinese Medicine and School of Pharmaceutical Sciences, Southern Medical University, Guangzhou, Guangdong 510515, China; Department of Emergency, Shenzhen People's Hospital, The First Affiliated Hospital, Southern University of Science and Technology, Shenzhen, Guangdong 518020, China; Department of Emergency, Shenzhen People's Hospital, The First Affiliated Hospital, Southern University of Science and Technology, Shenzhen, Guangdong 518020, China; School of Traditional Chinese Medicine and School of Pharmaceutical Sciences, Southern Medical University, Guangzhou, Guangdong 510515, China; Department of Emergency, Shenzhen People's Hospital, The First Affiliated Hospital, Southern University of Science and Technology, Shenzhen, Guangdong 518020, China; Department of Infectious Disease, Shenzhen People's Hospital, The First Affiliated Hospital, Southern University of Science and Technology, Shenzhen, Guangdong 518020, China; Department of Emergency, Shenzhen People's Hospital, The First Affiliated Hospital, Southern University of Science and Technology, Shenzhen, Guangdong 518020, China; School of Traditional Chinese Medicine and School of Pharmaceutical Sciences, Southern Medical University, Guangzhou, Guangdong 510515, China; State Key Laboratory for Quality Ensurance and Sustainable Use of Dao-di Herbs, Artemisinin Research Center, and Institute of Chinese Materia Medica, China Academy of Chinese Medical Sciences, Beijing 100700, China; State Key Laboratory of Antiviral Drugs, School of Pharmacy, Henan University, Kaifeng, Henan 475004, China

Sepsis, a life-threatening condition triggered by a dysregulated host response to infection, remains a major challenge for therapeutic intervention. Despite the growing interest in immunomodulatory strategies for sepsis treatment, the effects and mechanisms of these approaches on the organ-specific inflammatory and immunosuppressive states induced by sepsis are poorly understood. According to existing studies, capsaicin (CPS) has pharmacological effects such as analgesic, antipruritic, hypolipidemia, hypoglycemia, anti-inflammatory and antibacterial, and anti-tumor activity.^1^ However, the effectiveness of CPS in treating sepsis is still unknown. Here, we applied single-cell RNA sequencing (scRNA-seq) to reveal how CPS, a natural compound with anti-inflammatory properties, modulates the splenic microenvironment in a mouse model for sepsis induced by cecal ligation and puncture (CLP). We found that CPS improved survival and reduced inflammation in septic mice. Additionally, scRNA-seq analysis revealed that CPS plays an antioxidant and anti-inflammatory role in the body by regulating plasma cells derived from B cells. Moreover, CPS activated cytotoxic T cells and natural killer cells. Furthermore, we have observed that CPS has the potential to regulate cytokine production but exerts limited influence on the chemotactic activity of neutrophils during sepsis. Lastly, CPS enhanced the polarization of inflammatory macrophages during sepsis. Hence, our findings imply that CPS holds the potential to harmonize immune homeostasis, thereby dampening the inflammatory and/or immunosuppressive milieu observed in septic conditions. Our investigation presents a comprehensive scrutiny of CPS's role in orchestrating the spleen microenvironment in the context of septic infection, offering a foundational rationale for considering CPS as a prospective therapeutic intervention against sepsis.

To delineate the transcriptomic landscape and cellular composition of CPS efficacy, mice were divided into three groups including the Sham, the CLP model group, and the CPS group. At 4 h after surgery, the mice in the CPS group were injected intraperitoneally with 10 mg/kg of CPS solution, and the rest of the mice were injected with vehicle solution in equal amounts. Mice were executed and spleen tissue was removed 12 h later for scRNA-seq analysis or follow-up experiments, and the rest of the mice were observed for 96 h for survival rate analysis. A summary was provided for the entire research process in Figure 1A. We found that the mice in the CPS group had a better survival rate compared with the mice in the CLP group (Fig. S1A). Histopathological findings showed that CPS treatment improved the pathological changes of spleen tissue caused by CLP (Fig. S1B). Furthermore, sepsis increased the serum levels of pro-inflammatory factors (such as TNF-α, IL-1β, and IL-6) in the septic mice, while CPS treatment could effectively reduce the inflammatory storm (Fig. S1C–E).

**Figure 1:**
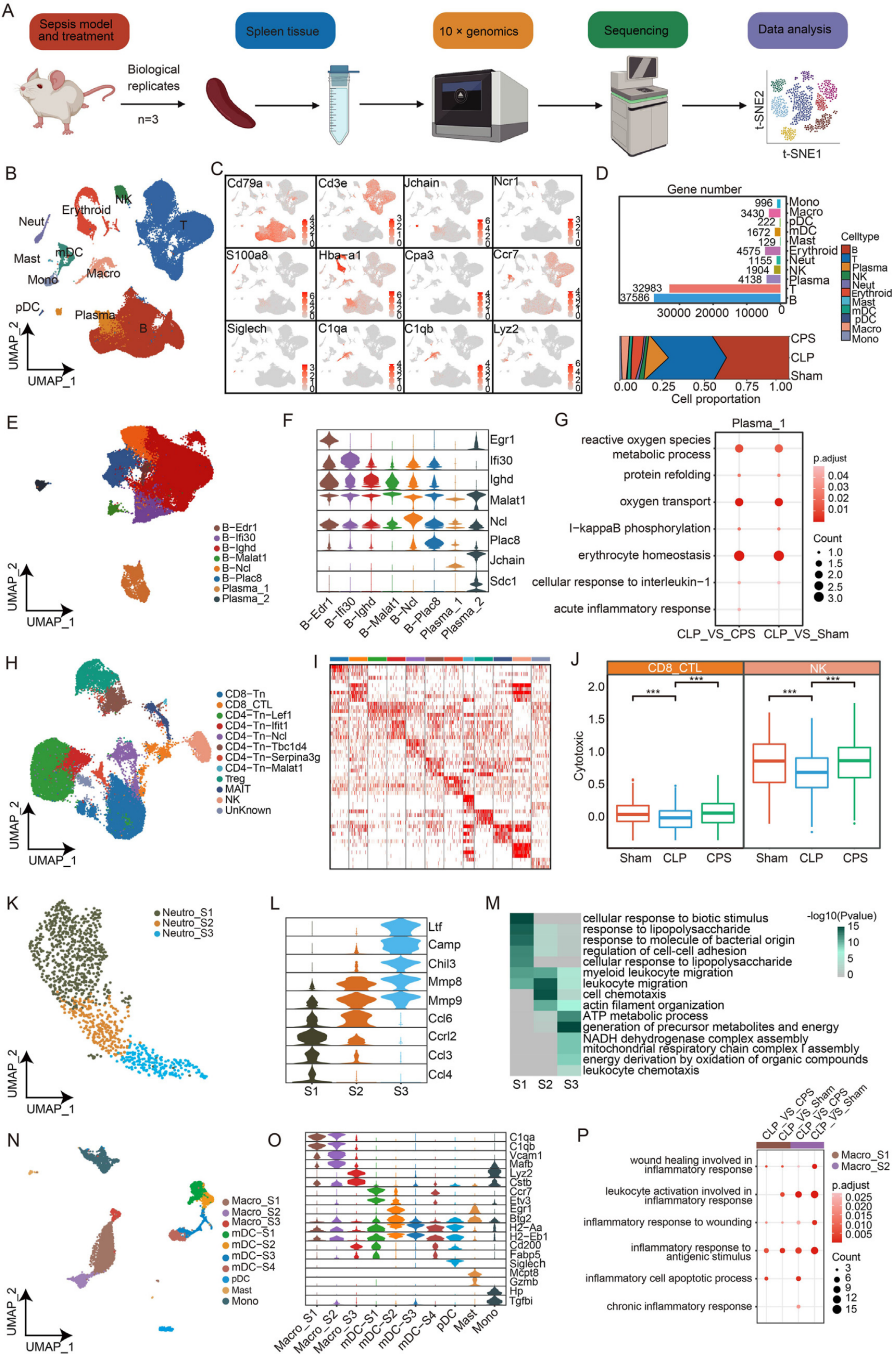
The transcriptomic signatures of single cell in mice. **(A)** The schematic workflow of the experiment design and analysis of the current study. **(B)** UMAP plot for 11 immune cell types. **(C)** UMAP visualization of the expression of canonical marker genes for 11 immune cell types. **(D)** The histogram of cell numbers for 11 immune cell types (top) and proportion changes of 11 immune cell types from each group (bottom). **(E)** UMAP plot for B cell (sub)types. **(F)** The violin plot showing the expression of canonical marker genes identified in B cell (sub)types. **(G)** The bubble pattern shows the up-regulated gene enrichment pathways in CLP versus Sham group or CLP versus CPS group in B cell (sub)types. **(H)** The UMAP visualization of unsupervised scRNA-seq clustering across T and NK cells. **(I)** The heat map showing the expression of canonical marker genes identified in T and NK cell (sub)types. **(J)** The box plots showing the module scores of cytotoxic pathways across CD8_CTL and NK in each group. **(K)** The UMAP visualization of unsupervised scRNA-seq clustering in neutrophil cells. **(L)** The violin plots showing the expression levels of the markers identified in neutrophil cell (sub)types. **(M)** The heatmap showing the enriched GO terms for each neutrophil (sub)type. **(N)** The UMAP visualization of unsupervised scRNA-seq clustering in myeloid cells. **(O)** The violin plots showing the expression of canonical marker genes identified in myeloid cell (sub)types. **(P)** The bubble pattern shows the up-regulated gene enrichment pathways in CLP versus Sham group or CLP versus CPS group across Macro_S1 and Macro_S2.

After quality control in scRNA-seq analysis, we obtained a total of 88,790 cells for further analysis (Fig. S1A). According to the expression levels of canonical marker genes,^2^ we identified 11 key cell types: erythroblast (*Hba-a1+*), T cell (*Cd3e+*) and B cell (*Cd79a*+), natural killer cell (*Nkg7+*), plasma cell (Plasma, *Jchain+*), monocyte (*Lyz+*), macrophage (Macro, *C1qb+*), neutrophil (Neutro, *S100a8+*), plasmacytoid dendritic cell (*Siglech+*), myeloid dendritic cell (mDC, *Ccr7+*), and mast cell (*Cpa3+*) (Fig. 1B, C; Fig. S2C). Furthermore, we depicted the cellular proportions of each cell type from the three groups (Fig. 1D). Compared with the Sham, part of the cell types returned to their original cellular compositions after CPS treatment, suggesting that the regulatory effect of CPS on septic spleen (Fig. 1D). We then evaluated the enrichment score related to inflammatory signaling across each cell type, including IL6_JAK_STAT3_SIGNALING in the three groups based on module scores estimation (Fig. S2D). The results suggested that most of the cells in the CLP group have higher module scores compared with those in the Sham group. We also noticed that the activation of module scores across most cells was significantly reduced after CPS treatment.

To further investigate the profile of B cells from sham-, CLP-, and CPS-treated mice, we re-clustered them into eight (sub)types based on the expression of representative marker genes, including B-Edr1, B-Ifi30, B-Ighd, B-Malat1, B-Ncl, B-Plac8, Plasma_1, and Plasma_2 (Fig. 1E, F). We counted the proportion of (sub)types of B cells in each group and found that the proportion of Plasma_1 cells remarkably increased in the CLP-induced spleen (Fig. S3A). According to previous studies, plasma cells are differentiated from mature B cells and secrete antibodies, which are a group of important immune cells in the body.^3^ Thus, we evaluated the enrichment score of inflammation and ROS for Plasma_1 across the three groups and found that the CLP group had the highest inflammatory scores compared with the Sham or CPS group, and the reactive oxygen species scores were significantly decreased after CPS administration (Fig. S3D). We next conducted differentially expressed genes (DEGs) between two comparisons: CLP *vs*. Sham and CLP *vs*. CPS. We also subjected these DEGs to enrichment analysis (Fig. S3B). From the results, we found pathways associated with inflammatory response, including the metabolic process of reactive oxygen species, protein refolding, and cellular response to interleukin-1, were up-regulated in the CLP group (Fig. 1G). On the other hand, pathways related to immune regulation, such as B cell-mediated immunity and lymphocyte-mediated immunity, show a down-regulation trend during sepsis (Fig. S3D). These results suggested that CPS treatment displayed anti-oxidant and anti-inflammatory functions in plasma cells for septic mice.

To further investigate the dynamic changes of immunoregulation, we re-clustered T cells and annotated to twelve lymphoid (sub)types across the Sham group, CLP group, and CPS group according to the expression levels of key marker genes, including CD8-Tn, CD8-Tn-Lef1, CD4-Tn-Ifit1, CD4-Tn-Ncl, CD4-Tn-Serpina3g, CD4-Tn-Malat1, MAIT, and unknown (Fig. 1H, I). We counted the proportion of the lymphoid T cells in each group and found that the proportion of cytotoxic-related cells CD8_CTL and natural killer cells were decreased in the septic spleen tissues and recovered by CPS for CD8_CTL (Fig. S4B). According to previous research, the cytotoxic ability of T cells and natural killer cells is an important effector mechanism of the immune system against viral infections and cancer.^4^ To further investigate the cytotoxic effects during sepsis, we analyzed the changes in a range of cytotoxic genes before and after CPS administration in a sepsis model. The results showed that many cytotoxin-related genes were expressed in CD8_CTL and specifically in natural killer cells compared with other (sub)types (Fig. 1J). We then evaluated the enrichment cytotoxic score across three (sub)types in the three groups based on module score estimation. The results suggested that the CLP group had lower module scores in three (sub)types compared with the CLP group, suggesting that the immunity of T cells was strengthened after CPS treatment.

To unravel the underlying mechanisms of CPS effects in sepsis mice, we categorized neutrophils into three unique subpopulations. This classification of these cells included three subtypes: Neutro_S1, Neutro_S2, and Neutro_S3 (Fig. 1I). We observed a decrease in the cellular abundance of Neutro_S1 in both the Sham and CPS groups compared with the CLP group, while the proportion of Neutro_S2 increased in the CLP group (Fig. S6A). Further, the proportion of Neutro_S3 was remarkably increased in the CPS, followed by the CLP, and the least in the Sham group. To investigate the respective functions of the neutrophil (sub)groups, we then performed functional enrichment analysis for each (sub)type. The results showed that pathways involved in the cellular response to biotic stimulus and lipopolysaccharide were more prominent in Neutro_S1. The function of Neutro_S2 is prominent in cell chemotaxis and leukocyte migration, while the function of Neutro_S3 was more biased towards mitochondrial respiratory chain complex I assembly, energy derivation by oxidation of organic compounds, and NADH dehydrogenase complex assembly (Fig. 1M). Thus, we supposed that CPS could regulate CLP-induced neutrophil differentiation and metabolic energy.

Myeloid cells were the significantly altered cell type in CLP, indicating distinct immune responses. Those cells were re-clustered to annotate their transcriptional features (Fig. 1H). Ten cell subsets were annotated with canonical marker genes, including Macro_S1, Macro_S2, Macro_S3, mDC-S1, mDC-S2, mDC-S3, mDC-S4, plasmacytoid dendritic cells, mast cells, and monocytes (Fig. 1I). We discovered that within these (sub)types, the Macro_S1 subtype exhibited characteristics of M2-like macrophages, marked by *Cd86*, while the Macro_S3 subtype displayed traits of M1-like macrophages, indicated by the presence of the marker *Mrc1*.^5^ While Macro_S2 was identified by the M1-like and M2-like marker genes, which implied that Macro_S2 was in the middle state (Fig. S5C). To describe the transcriptomic profile changes of the Macro_S1 cells, Macro_S2 cells, and Macro_S3 cells among the Sham group, the CLP group, and the CPS group, we explored DEGs of CLP *vs*. Sham and CLP *vs*. CPS, separately. Data analysis revealed that Macro_S3 had few DEGs among the three groups (Fig. S5B). We further subjected Macro_S1 and Macro_S2 DEG GO enrichment analysis. Our results showed that several biological pathways, including response to wound healing involved in inflammatory response, inflammatory response to antigenic stimulus, and chronic inflammation were activated during sepsis infection, which could be restored to baseline levels after CPS treatment (Fig. 1P).

Our investigation applied scRNA-seq in exploring the modulation of immune cells in the septic spleen. This endeavor offers new insights and methods for consideration in the clinical management of sepsis. Although we have detected the regulatory effects of CPS, there are still some limitations. Regrettably, CPS can cause a certain irritation to the skin if using subcutaneous injection. New methods like nanotechnology and biochemistry are expected to be developed in the future for the widespread use of CPS.

In summary, this study combines biophysical experiments and single-cell sequencing technology to investigate the potential mechanism of CPS in treating sepsis and modulating immune cells. Our results indicate that CPS can globally regulate the CLP-induced immunosuppression in sepsis, thus broadening its clinical utility and offering a novel insight for its therapeutic application.

## Ethics declaration

All research procedures were approved by the Institutional Review Board of the Shenzhen People's Hospital and conducted in accordance with the Declaration of Helsinki.

## Conflict of interests

The authors have declared that they have no conflict of interests.

## Funding

This work was supported by the Establishment of Sino-Austria “Belt and Road” Joint Laboratory on Traditional Chinese Medicine for Severe Infectious Diseases and Joint Research (No. 2020YFE0205100), the 10.13039/501100012166National Key Research and Development Program of China (No. 2020YFA0908000), the Innovation Team and Talents Cultivation Program of the National Administration of Traditional Chinese Medicine (China) (No. ZYYCXTD-C-202002), the 10.13039/501100001809National Natural Science Foundation of China (No. 82074098, 81841001), the Fundamental Research Funds for the Central Public Welfare Research Institutes (China) (No. ZZ13-ZD-07, ZXKT18003, ZZ14-YQ-050, ZZ14-ND-010, ZZ15-ND-10, ZZ14-FL-002, ZZ14-YQ059, ZZ15-YQ-063), Shenzhen Science and Technology Innovation Commission (Guangdong, China) (No. JCYJ20210324115800001 and JCYJ20210324114014039), the National Key R&D Program of China Key projects for international cooperation on science, technology and innovation (No. 2020YFE0205100), the 10.13039/501100004791Shenzhen Medical Research Fund of 10.13039/501100004791Shenzhen Medical Academy of Research and Translation (Guangdong, China) (No. B2302051), the Distinguished Expert Project of Sichuan Province Tianfu Scholar (China) (No. CW202002), 10.13039/501100005892China Academy of Chinese Medical Sciences (10.13039/501100005892CACMS) Innovation Fund (No. CI2023E002), and State Key Laboratory for Quality Ensurance and Sustainable Use of Dao-di Herbs.

## Figures and Tables

**figs1:**
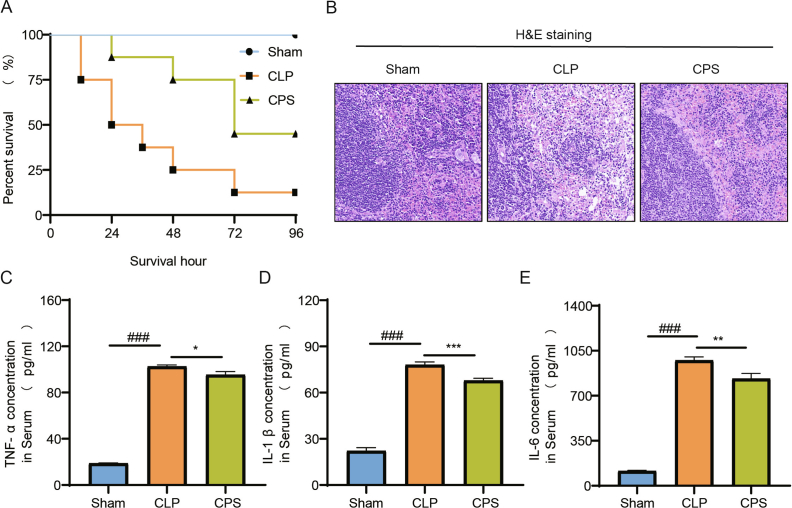


**figs2:**
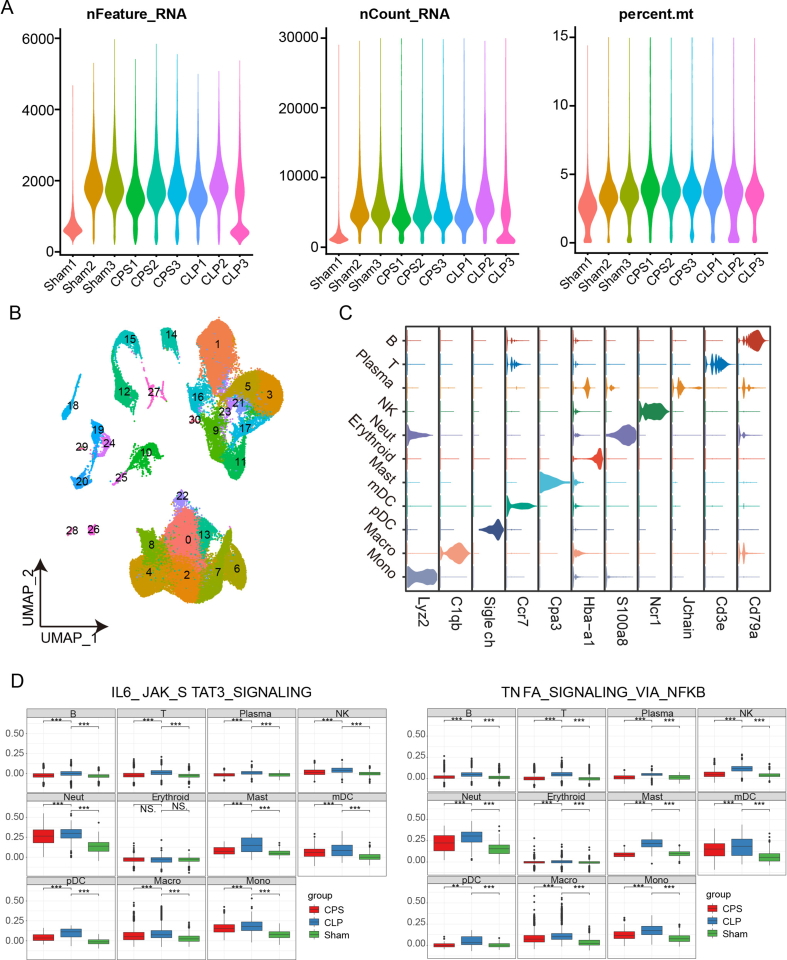


**figs3:**
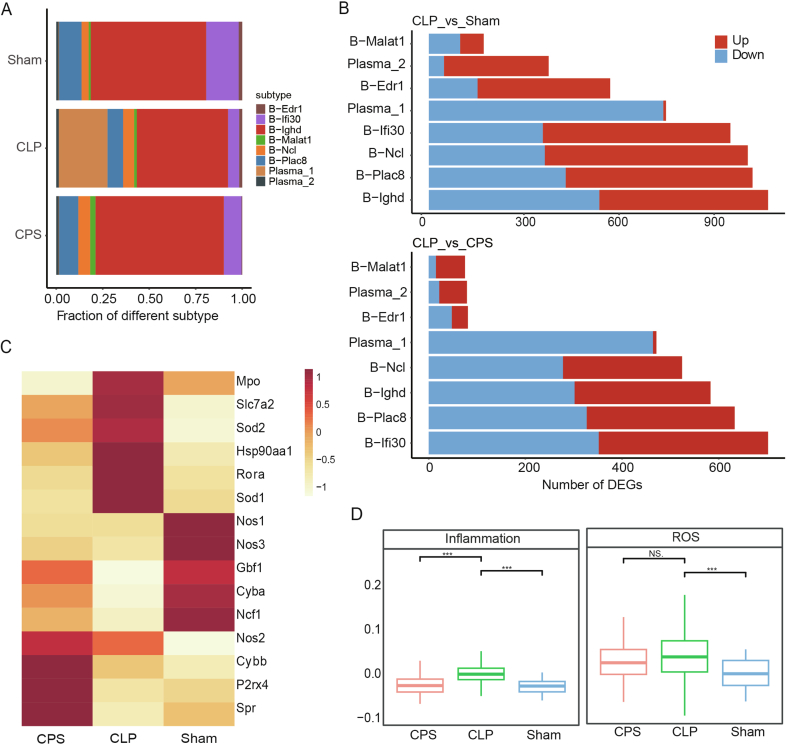


**figs4:**
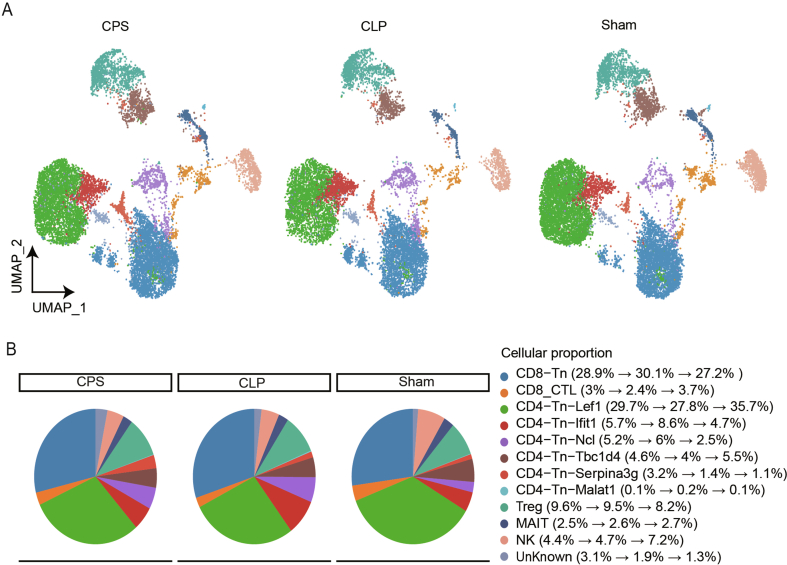


**figs5:**
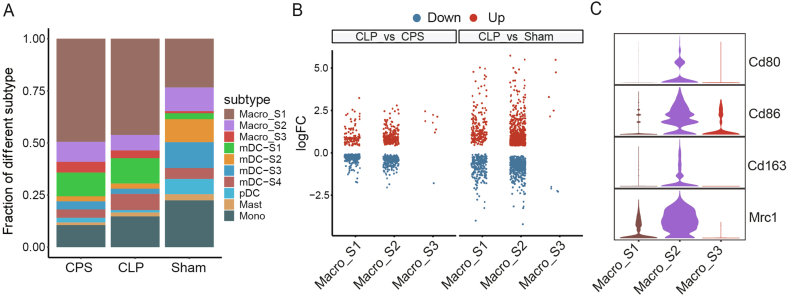


**figs6:**