# Supplementary material for: Single-cell transcriptomics illustrates the immune inflammatory responses of septic mice spleen after capsaicin treatment
Source: Genes Dis. 2024 Mar 5;12(1):101256. doi: 10.1016/j.gendis.2024.101256 (PMC11456796; doi:10.1016/j.gendis.2024.101256)
Supplement: Multimedia component 1 [file mmc1.pdf]

# Single-cell transcriptomics illustrates the immune inflammatory responses of septic mice spleen after capsaicin treatment

Weijin Qiu[1], Qian Zhang[1], Jing Liu[1], Xueling He[1], Guangqing Cheng[3], Jiayun Chen[2], Yunmeng Bai[3], Piao Luo[1], Ping Gong[3], Fei Shi[3], Jigang Wang[2,*]

## Supplementary Information Containing:

1. Methods
2. Additional Results
3. Discussion
4. References
5. Availability of data and material
6. Supplementary Figures

# Supplementary Information

## 1. Methods

### 1.1 Mice

Healthy BALb/c mice were purchased from the Beijing Vital River Laboratory Animal Technology Co., Ltd. Mice (male, 18-22g, 6-8 weeks), were kept in Specified Pathogen Free barrier environment with drink and eat freely. Our experimental protocols were approved by Institutional Animal Care and Use Committee (IACUC) of the Institute of Institute of Traditional Chinese Medicine, China Academy of Chinese Medical Sciences.

### 1.2 Sepsis model and CPS treatment

Mice were divided into three groups including the Sham group, the CLP group, and CPS group at the end of the adaptive feeding. The sepsis model was established as described previously[1]. At 4 h after surgery, the CPS group was injected intraperitoneally with 10 mg/kg of CPS solution, and the rest of the mice were injected with vehicle solution in equal amounts. Mice were executed and spleen tissue was removed 12 h later for scRNA-seq analysis or follow-up experiments. Mice were observed for 96-hour survival studies.

### 1.3 Hematoxylin-eosin staining

The spleen was fixed in 4% paraformaldehyde, then the spleen was dehydrated, paraffin-embedded, and cut into thin slices of 4 μm thickness. Sections were dewaxed in xylene solvent, dehydrated in a series of ethanol, stained with eosin-hematoxylin solution, rehydrated in a series of ethanol, and finally sealed. Histological images were observed and photographed under a microscope.

## 1.4 Enzyme Linked Immunosorbent Assay (ELISA)

Inflammatory factors in mouse plasma by ELISA kit (TNF-α, Mlbio China YJ002095; IL-1β, Mlbio China, YJ301814 and IL-6 Mlbio, China, YJ06159). Experiments are performed under the instructions supplied by the reagent manufacturer.

## 1.5 Isolation of splenic single cell suspensions

Briefly, the mouse spleens were lightly ground into single cells in a screen and then passed through a 40μM screen. Red blood cells (RBC) were removed with RBC lysis solution, and the concentration of cells was adjusted to 1000 cells/μl after twice washing in PBS solution. Finally, splenic single cells were loaded on the 10x Genomic Chromium Single Cell Controller For follow-up sequencing.

## 1.6 Chromium single-cell 3' library construction and sequencing

In the present study, the preparation of scRNA-seq libraries was executed as per the established protocols, aligning with methodologies detailed in prior publications. The process utilized the Chromium Single Cell 3′Library & Gel Bead Kit (PN-1000121, 10× Genomics, CA, USA), capturing approximately 10,000 cells per droplet to create single-cell gel bead emulsions (GEMs). These GEMs underwent reverse transcription as part of a PCR amplification process. Post reverse transcription and cell barcoding, emulsions were disrupted to isolate and purify the cDNA. This purification employed a cleaning mixture incorporating DynaBeads and SPRIselect reagents (B23318, Beckman Coulter, CA, USA), facilitating subsequent PCR amplification. The resultant amplified cDNA was integral to the construction of RNA-seq libraries, involving fragmentation, end repair, bidirectional size selection, and PCR amplification with indexing primers. Adhering strictly to the manufacturer's guidelines, the prepared libraries underwent purification and rigorous quality checks.

Finally, scRNA-seq data acquisition was carried out on an Illumina NovaSeq 6000 sequencer (Illumina, CA, USA), utilizing 150bp paired-end reads.

## 1.7 scRNA-seq dataset pre-treatment

The raw data underwent processing using the CellRanger 3.0 pipeline (10x Genomics), followed by further analysis including secondary clustering and differential expression using Seurat/R. Prior to clustering, all libraries and subsets were scrutinized for quality assurance. Cells displaying elevated levels of mitochondrial markers, indicative of lower quality, were excluded from each library/subset in the preparatory phase for downstream analysis. Subsequently, each library was globally scaled and normalized, applying a scale factor of $1\times10^4$ and a logarithmic transformation. Additionally, to enhance the accuracy of downstream analyses, dimension reduction, and UMAP projection for single-cell RNA sequencing (scRNA-seq) visualization, we meticulously identified and mitigated unwanted variabilities arising from biological noise and batch effects.

## 1.8 Alignment and UMI counting

The samples underwent several processing steps using the Cell Ranger Software Suite (v6.1.1) to handle de-multiplexing, barcode processing, and single-cell 3' UMI counting. For alignment of the sequencing reads in FASTQ format, the software employed STAR,[2] which aligned the reads to the mouse mm10 reference genome obtained from Ensembl. The identification of cell barcodes was done based on the distribution of UMI counts, and this step was performed automatically. Finally, to remove any batch effects, the gene-barcode matrices from all six samples were integrated using Seurat.

## 1.9 Quality control

Each sample underwent rigorous quality control measures to determine the

appropriate threshold. The gene-barcode matrix, generated by Cell Ranger, was imported into R as a Seurat (v4.0.3) object to facilitate subsequent steps including filtering, data normalization, dimension reduction, clustering, and gene differential expression analysis. Genes were excluded from the analysis if they were detectable in fewer than three cells. Subsequently, specific criteria were applied to each cell, requiring a gene count between 200 and 6000, UMI count between 500 and 30000, and a mitochondrial gene percentage below 0.15. Following this meticulous filtering process, cells remained for further analysis.

## 1.10 Data normalization and integration

We employed SCTransform to normalize individual samples and identify highly variable genes. Additionally, to mitigate any potential influence of batch effects arising from varying mice conditions, we implemented a data integration methodology utilizing the Seurat framework on the SCTransform-normalized data. Specifically, we utilized the PrepSCTIntegration function to identify anchors, and set the normalization. Method parameter to 'SCT' during the execution of FindIntegrationAnchors and IntegrateData. This resulted in the generation of an integrated dataset encompassing six samples.

## 1.11 Dimensionality reduction, clustering, and cell type identification

The consolidated gene-barcode matrix underwent principal component analysis (PCA) using all the identified variable genes. This was followed by the construction of a shared nearest neighbor graph, based on Euclidean distances within the lower-dimensional space defined by the significant principal components (dimensions 1-43). Subsequently, cells were grouped into clusters at a suitable resolution setting (resolution=0.15) and visualized using a two-dimensional Uniform Manifold Approximation and Projection (UMAP) algorithm for Dimension Reduction. To identify differential expression of genes (DEGs) within each cluster, we employed the

Wilcoxon rank-sum test. Cell types were determined using well-established marker genes. For specific cell types, clustering was executed based on these markers, and the resultant clusters were analyzed using Seurat v3 in a manner analogous to the broader analysis.

## 1.12 Differential genes expression analysis

We eliminated cell types with incomplete or less than 25% representation in the comparison groups before conducting the differential expression analysis. Differential gene expression analysis was performed for each cell type using the Seurat function FindAllMarkers, employing the Wilcoxon rank-sum test. Differential genes between two groups were identified using the following criteria: $|LogFC| > 0.1$ and adjusted p-value $< 0.05$. To visualize the markers, violin plots or heatmaps were generated using the R packages MySeuratWrappers (v0.1.0) and pheatmap (v1.0.12).

## 1.13 Gene functional enrichment analysis

Gene ontology (GO) was conducted using the R package clusterProfiler (v4.0.2) to assess the differentially expressed genes (DEGs) in T cell (sub)types, B cell (sub)types, Neutrophil cell (sub)types, and Myeloid cell (sub)types.[3] To account for multiple hypothesis testing, the Benjamini-Hochberg procedure was employed for correction. Furthermore, for in-depth pathway analysis, we employed the R package GSVA (version 1.40.0), utilizing MSigDB Hallmark gene sets to identify significant pathways. Differential gene sets were calculated using the R package limma (version 3.48.1), particularly in cell type analyses[4]. Only those results yielding adjusted p-values less than 0.05 were selected for further examination. The visualization and detailed analysis of these filtered results were executed using the R package ggplot2 (version 3.3.5), enabling a comprehensive and illustrative representation of the data.

### 1.14 Statistical analysis

The results in this study are presented as the mean ± SEM of the mean. One-way ANOVA was used to analyze the statistical differences between three groups. Differences between the groups were considered significant at $P < 0.05$ (# Sham group VS CLP group, $P < 0.05$; ## Sham group VS CLP group, $P < 0.05$; ### Sham group; *CLP group VS CPS group, $P < 0.05$; ## CLP group VS CPS group, $P < 0.05$; ### CLP group VS CPS group), and ns indicated not significant.

## 2. Additional Results

### 2.1 Transcriptional heterogeneity exists within CLP-induced sepsis mice and CPS treatment mice

After single-cell preparation of Mice spleen tissue, approximately 20000 cells per sample were loaded onto the 10× Genomics platform for scRNA-seq. Approximately 30000 cells per sample were recovered from the sequencing data via the standard Cell Ranger protocol. The detailed filtration parameters and the specific sample information were mentioned in the method. After strict quality control, a total of 88790 cells (32575 for CPS, 27770 for CLP, and 28445 for Sham) were obtained for subsequent analysis **(Fig. S2A)**. These cells were integrated and divided into thirty-one different subsets. **(Fig. S2B)**. Based on the expression levels of canonical marker genes, we cluster those cell types into 11 major cell types. **(Fig. S2C)**.

### 2.2 CPS suppressed the activation of splenic B cells in mice with sepsis.

We counted the proportion of (sub)types of B cells in each group and found that the number of B cell (sub)types did not change regularly before and after CPS

administration in septic spleen tissue **(Fig. S3A)**. In B cells, the proportion of most B cell (sub)types in the Mode group was down-regulated compared with those in the CPS group and Sham group. Conversely, (sub)types of cells with Plasma were up-regulated in the Model compared to the other two groups **(Fig. S3A)**. In addition, we profiled the up-regulated DEG patterns of Sham vs CLP groups. and CLP vs CPS groups among B cell (sub)types, finding that most activated DEGs were down-regulated after CPS treatment **(Fig. S3B)**.

To investigate the other functions of B cells, we analyzed the changes in a range of representative genes before and after CPS administration in a sepsis model. Overall, compared to the Sham group and the CLP group, the CPS group showed enhanced expression of genes like Nos2, Cybb, P2rx4, and Spr. Furthermore, compared to the CPS group and the CLP group, the Sham group showed enhanced expression of genes like Nos1, Nos3, Gbf1, Ncf1, and Cyba. Finally, compared to the CPS group and the Sham group, the CLP group showed enhanced expression of genes like Mpo, Slc7a2, Sod2, Hsp90aa1, Rora, and Sod1.

## 2.3 CPS impacts the immune function and reactive oxygen species production in neutrophils.

To investigate the immunomodulatory impact of CPS on neutrophils during sepsis, we conducted a detailed analysis of the expression levels of neutrophil effector genes across the three groups. This approach was aimed at elucidating the intricate interactions and potential regulatory mechanisms influenced by CPS in the context of sepsis-related immune responses **(Fig. S6B)**. We found that compared with the Sham group, immunoregulation genes including Il10rb, Tnf, and Tlr2 were upregulated in sepsis, while CPS could inhibit the expression of these genes. In addition, CPS was able to down-regulate the expression level of Cd274 (PD-L1) genes while having no evident effect on MHC-II components (H2-Ab1, H2-Aa) of neutrophils. We also found that the expression of chemotaxis genes including Ccr1 and Cxcr2 were

downregulated in sepsis but restored to baseline after CPS treatment. Furthermore, GO analysis based on the CLP vs Sham and CLP vs CPS DEGs revealed that sepsis could upregulate response to inflammatory regulation, cytokine-mediated signaling pathway, and the toll-like receptor signaling pathway in Neutro_S1 and Neutro_S2 (sub)type, while CPS treatment could reverse these enriched pathways, indicating the anti-inflammatory effect of CPS on neutrophils **(Fig. S6C)**. Previous studies have reported that sepsis-induced oxidative stress can lead to the infiltration of neutrophil inflammatory reactions.[5] Therefore, we evaluated the module scores of REACTIVE_OXYGEN_SPECIES_PATHWAY of neutrophils across three groups, finding that neutrophils in the CLP group manifested a higher module score compared with other groups **(Fig. S6D)**. As for the gene expression patterns of the reaction oxygen species (ROS) in Neutro_S1 and Neutro_S2 (sub)types, we found that most of ROS-associated genes were activated in the CLP group and down-regulated after CPS treatment **(Fig. S6E)**.

## 3. Discussion on additional results

While CPS has been recognized for its potential anti-inflammatory and therapeutic pharmacological properties against sepsis in previous studies, [6,7] the precise underlying mechanism has remained elusive. In our investigation, we observed that mice receiving CPS treatment displayed remarkable resilience against CLP-induced sepsis when compared to the Sham group. In addition, mice in the CPS group exhibited improved splenic pathological features and diminished inflammation. Although splenic immune cell subsets did not vary significantly between groups, CPS could significantly inhibit inflammatory pathway (IL-6_JAK_STAT3_SINGNALING and TNFA_SINGNALING) activation.

B cells represent a crucial subset of lymphocytes, serving as key mediators in both adaptive and innate immune responses. They accomplish this through various

means, such as antibody production, antigen presentation, and cytokine secretion.[8] In the context of sepsis and inflammation, B cells engage in intricate interactions with other innate and adaptive immune cells, exerting mutual influences on each other's functions. [9] Among lymphocytes, regulatory B cells emerge as a specialized subset with notable immunomodulatory roles. These cells assume a vital role in preserving immune homeostasis and fostering tolerance by generating anti-inflammatory cytokines and suppressing inflammatory responses. [10] In addition to changes in the expression of ROS and inflammatory pathways, we also looked at some representative genes in some B cells. Strangely, they were all highly expressed in their respective samples, and their role in the treatment of sepsis needs to be studied.

Macrophages are a crucial component of the host's primary defense mechanism. M1 and M2 macrophages, each with distinct functions, possess the unique capability to either destroy pathogens or repair inflammation-related injuries. It is widely recognized that the balance in M1/M2 macrophage polarization plays a pivotal role in determining the outcome of organs subjected to inflammation or injury. In instances of severe infection or inflammation impacting an organ, macrophages initially adopt an M1 phenotype, releasing pro-inflammatory factors to combat the stimulus.[12] However, prolonged activation of the M1 phase can lead to tissue damage. In contrast, M2 macrophages play a restorative role by secreting significant levels of IL-10 and TGF-β, which not only suppress inflammation but also aid in tissue repair, remodeling, revascularization, and the maintenance of homeostasis.

n both animal models and patients experiencing the immunosuppressed phase of sepsis, we observed that the abundant presence of Pathogen-Associated Molecular Patterns (PAMPs) precipitates a significant alteration in the state of neutrophils. This finding underscores the profound impact of PAMPs on neutrophil behavior during this critical phase of sepsis, highlighting a key aspect of the host-pathogen interaction in both experimental and clinical settings. This altered state is characterized by impaired bacterial clearance, reduced reactivity, diminished reactive oxygen species (ROS)

production, and a notable decrease in the recruitment of neutrophils to infected tissues [13, 14]. In our study, we have observed that CPS has the potential to regulate cytokine production but exerts limited influence on the chemotactic activity of neutrophils during sepsis. Furthermore, the inhibitory effects of CPS on both the Toll-Like Receptor (TLR) signaling pathway and cytokine-mediated signaling pathways reflect its anti-inflammatory impact on neutrophils. Additionally, the down-regulation of ROS pathways and associated genes further underscore the antioxidative effects of CPS on neutrophils in the context of sepsis.

Currently, understanding the intricate regulatory effects of CPS on immune cells in sepsis remains a complex challenge. Regrettably, this study does not provide conclusive information regarding the specific target of CPS in sepsis treatment. Identifying such targets may necessitate the utilization of alternative techniques, such as activity-based protein profiling (ABPP) [15], cellular thermal shift assay (CETSA) [16], and similar approaches. Nonetheless, our present investigation has leveraged single-cell RNA sequencing (scRNA-seq) to explore the modulation of immune cells in the spleen during sepsis by CPS. This endeavor offers fresh insights and avenues for consideration in the clinical management of sepsis.

## 4.Ethics declaration

All research procedures were approved by the Institutional Review Board of the Shenzhen People's Hospital and conducted in accordance with the Declaration of Helsinki.

## 5. Availability of data and material

The raw single-cell RNA sequencing (scRNA-seq) datasets utilized in this study are accessible upon reasonable request directed to the corresponding author. This

provision ensures that interested researchers can engage with the primary data underpinning our findings, subject to standard academic and ethical protocols.

## References


1 Yan Z, Luo H, Xie B, Tian T, Li S, Chen Z*, et al*. Targeting adaptor protein SLP76 of RAGE as a therapeutic approach for lethal sepsis. Nat Commun 2021; 12: 308.

2 Dobin A, Davis CA, Schlesinger F, Drenkow J, Zaleski C, Jha S*, et al*. STAR: ultrafast universal RNA-seq aligner. Bioinformatics 2013; 29: 15-21.

3 Yu G, Wang LG, Han Y, He QY. clusterProfiler: an R package for comparing biological themes among gene clusters. OMICS 2012; 16: 284-7.

4 <GSVA gene set variation analysis fo Source BMC Bioinformatics SO 2013 Jan 16 14 7.PDF>.

5 Yao RQ, Li ZX, Wang LX, Li YX, Zheng LY, Dong N, et al. Single-cell transcriptome profiling of the immune space-time landscape reveals dendritic cell regulatory program in polymicrobial sepsis. Theranostics 2022; 12: 4606-28.

6 Srinivasan K. Biological activities of red pepper (Capsicum annuum) and its pungent principle capsaicin:a review[J].Critical Reviews in Food Science and Nutrition.2015.

7 Sharma S.Mechanisms and clinical uses of capsaicin[J].European Journal of Pharmacology,2013,720(1/3):55-62.

8 Shen P, Fillatreau S. Antibody-independent functions of B cells: a focus on cytokines. Nat Rev Immunol 2015; 15: 441-51.

9 Dong X, Tu H, Qin S, Bai X, Yang F, Li Z. Insights into the Roles of B Cells in Patients with Sepsis. J Immunol Res 2023; 2023: 7408967.

10 Dasgupta S, Dasgupta S, Bandyopadhyay M. Regulatory B cells in infection, inflammation, and autoimmunity. Cell Immunol 2020; 352: 104076.

11 Murray PJ, Wynn TA. Protective and pathogenic functions of macrophage subsets. Nat Rev Immunol 2011; 11: 723-37.

12 Shapouri-Moghaddam A, Mohammadian S, Vazini H, Taghadosi M, Esmaeili SA, Mardani F, et al. Macrophage plasticity, polarization, and function in health and disease. J Cell Physiol 2018; 233: 6425-40.

13 Zmijewski JW, Pittet JF. Human Leukocyte Antigen-DR Deficiency and Immunosuppression-Related End-Organ Failure in SARS-CoV2 Infection. Anesth Analg 2020; 131: 989-92.

14 Winkler MS, Rissiek A, Priefler M, Schwedhelm E, Robbe L, Bauer A, et al. Human leucocyte antigen (HLA-DR) gene expression is reduced in sepsis and correlates with impaired TNFalpha response: A diagnostic tool for immunosuppression? PLoS One 2017; 12: e0182427.

15 Roberts AM, Ward CC, Nomura DK. Activity-based protein profiling for mapping and pharmacologically interrogating proteome-wide ligandable hotspots. Curr Opin Biotechnol 2017; 43: 25-33.

16 Mateus A, Kurzawa N, Perrin J, Bergamini G, Savitski MM. Drug Target Identification in Tissues by Thermal Proteome Profiling. Annu Rev Pharmacol Toxicol 2022; 62: 465-82.

# Supplementary Figures

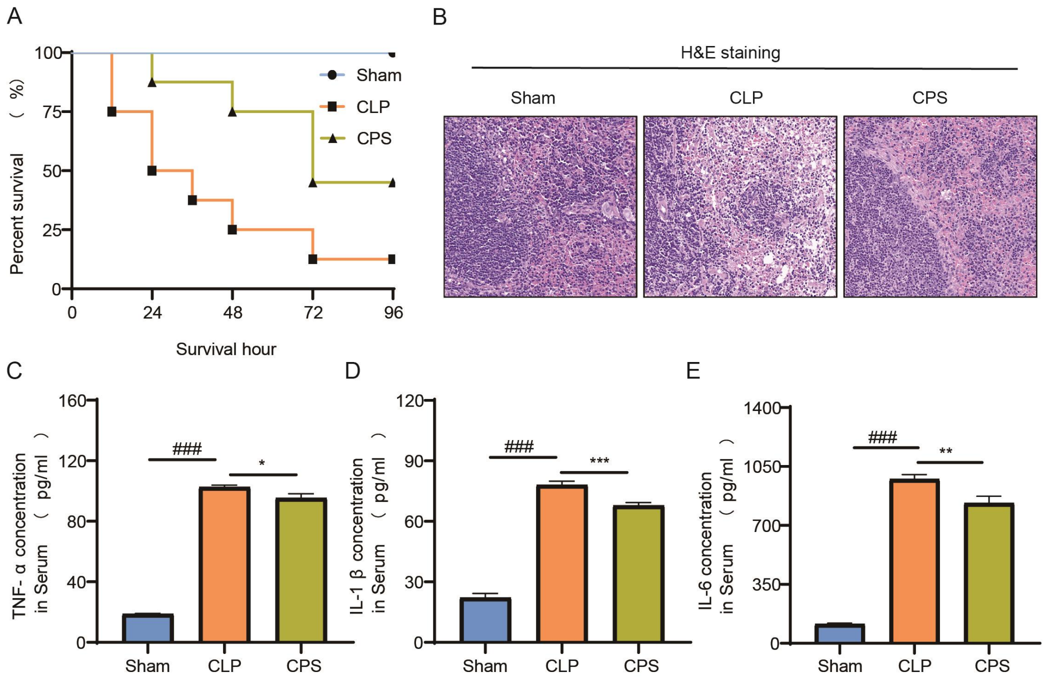


**Figure S1. Excellent effect of capsaicin on CLP-induced sepsis mice.** (A) The survival rate of septic mice in each group was recorded over 96h (n = 8); (B) H&E staining diagram of Spleen tissue in each group; (C) Relative expression level of TNF-α in serum; (D) Relative expression level of IL-1β in serum; (E) Relative expression level of IL-6 in serum.

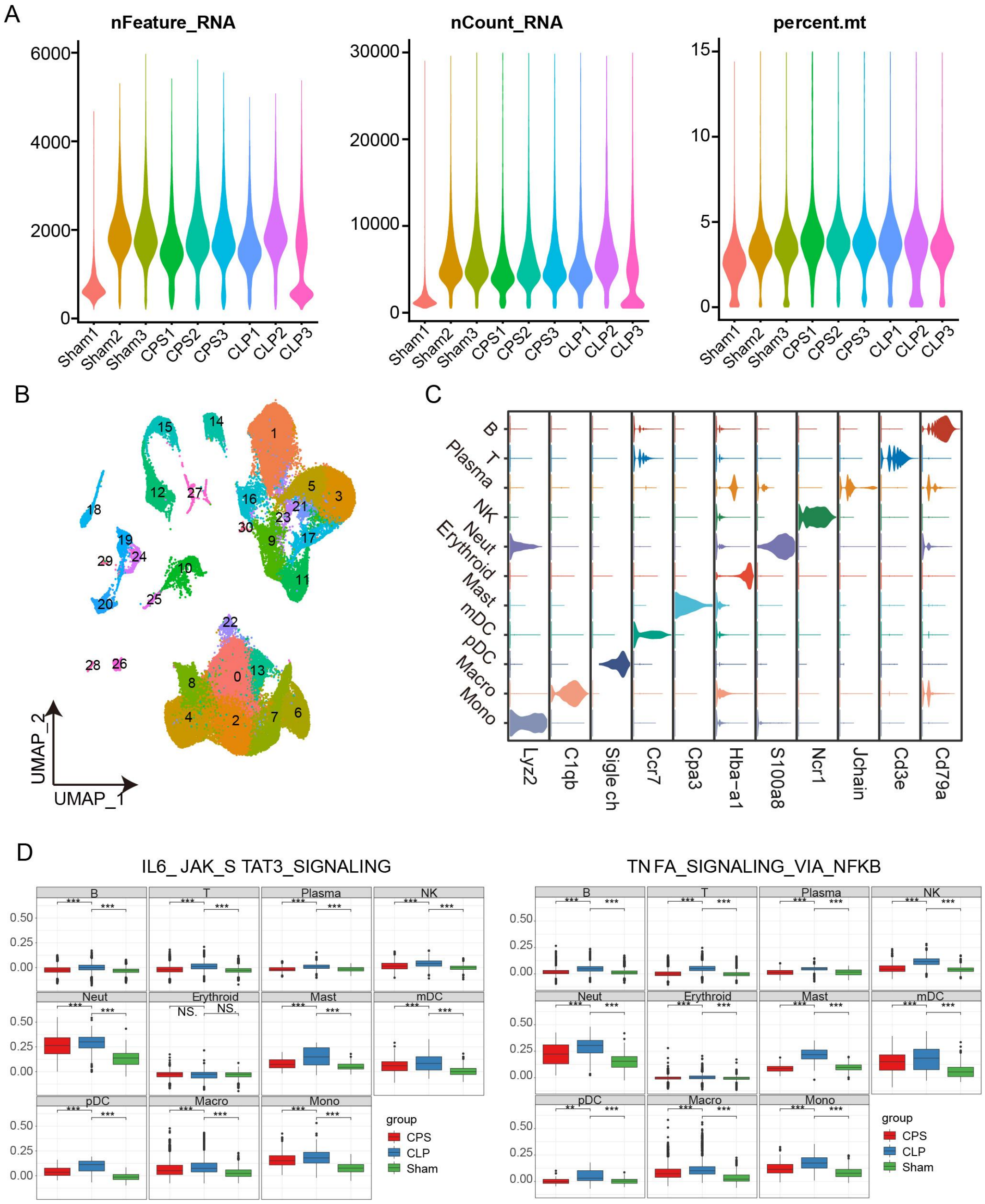


**Figure S2. Single-cell profiling of cellular landscape of spleen in sepsis and CPS-treatment.** (**A**) Distribution of nFeature_RNA (number of genes for each cell), nCount_RNA (number of transcripts for each cell) and percent.mt (percentage of mitochondrial genes for each cell) for every sample. (**B**) UMAP plot for Thirty-One cell types. (**C**) The violin plot depicted the expression levels of the respective selected markers across 12 clusters. (**D**) The box plots show the module scores of and IL6_JAK_STAT3_ SIGNALING (right panel) and TNFA_SIGNALING_VIA_NKFB pathways (down panel) across 11 cellular identities.

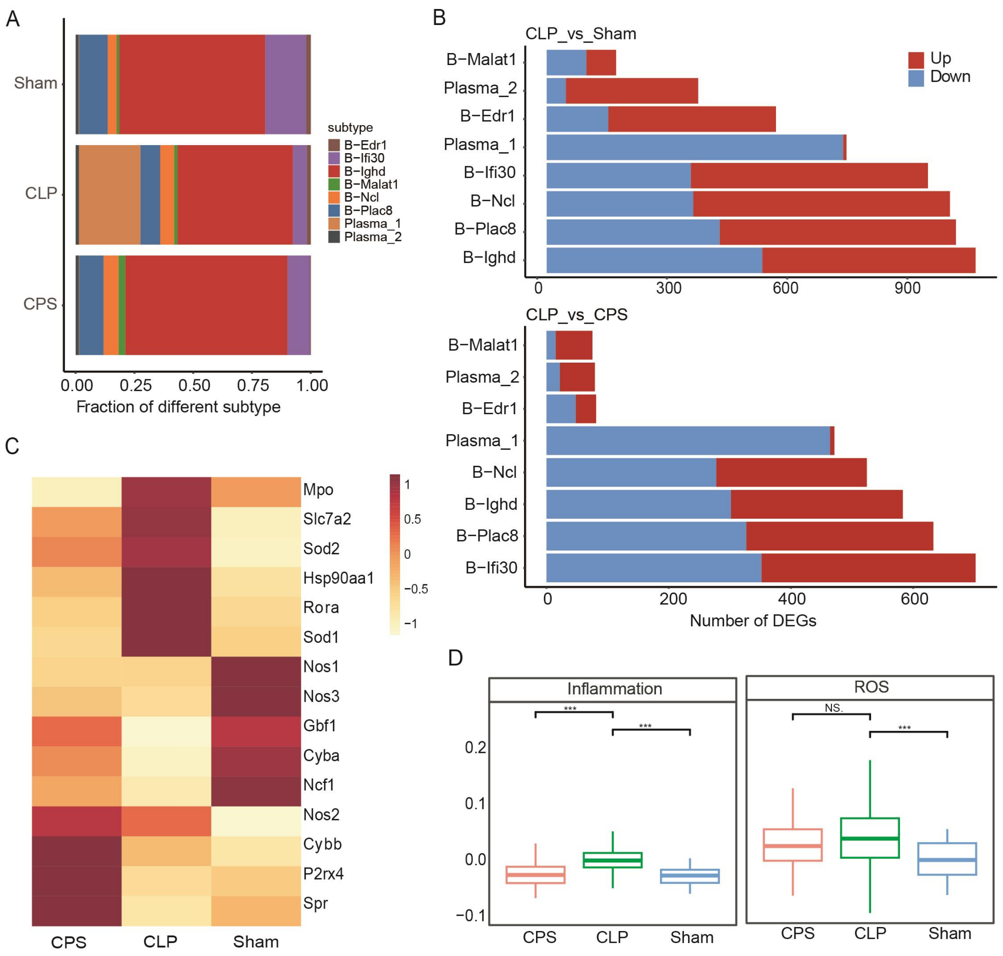


**Figure S3. CPS suppressed the activation of splenic B cells in mice with sepsis.** (**A**) The histogram shows the cellular proportion among 8 types, colored according to cell types. (**B**) The bar chart showed DEGs of each (sub)type in CLP vs Sham group (up panel) and CLP vs CPS group (down panel). (**C**) The heatmap plot depicts representative genes in B cell (sub)types across three groups. (**D**) The violin plot shows regulatory modules scores of Inflammation (left) and ROS pathways (right) for Plasma_1.

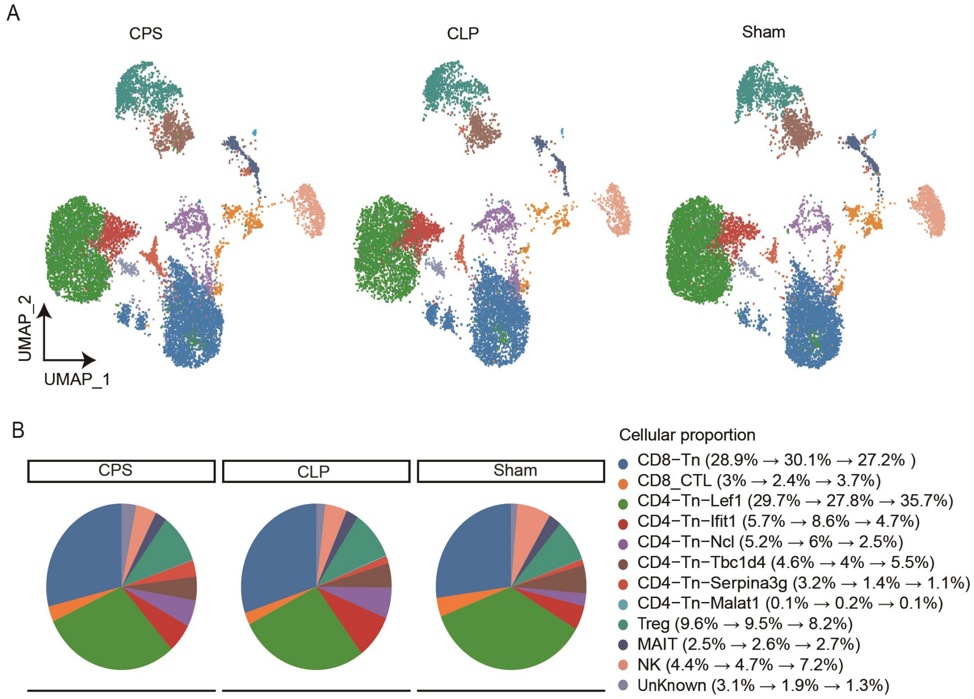


**Figure S4. CPS enhances the immune function of T cells in septic spleen.** (**A**) Three UMAP visualization show unsupervised scRNA-seq clustering, revealing ratio of 12 distinct cellular identities from each group, respectively. (**B**) The pie plot shows the cellular proportion among seven T lymphocytes and NK (sub)types in each group, colored according to (sub)types.

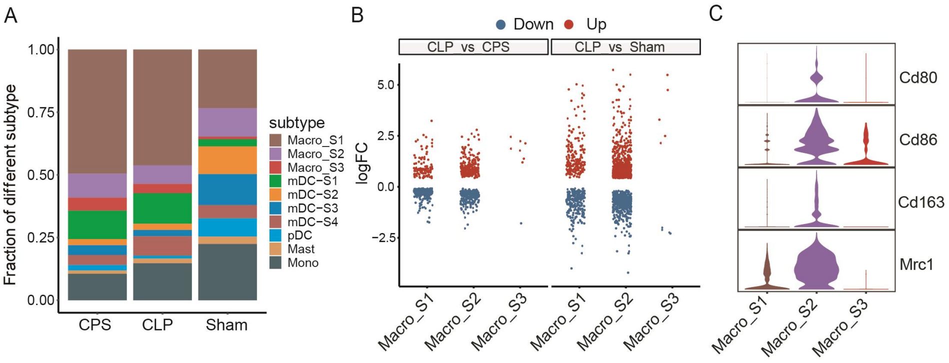


**Figure S5. CPS regulates macrophage polarization and DC cell fate in CLP-induce sepsis model**. (**A**) The histogram shows the cellular proportion among 8 types, colored according to cell types. (**B**) Strip chart showing DEGs of three groups in Macro_S1, Macro_S2 and Macro_s3. (**C**) The violin plot shows the relative expression levels of M1-like and M2-like markers of each group across Macro_S1, Macro_S2 and Macro_S3 in scRNA-seq datasets.

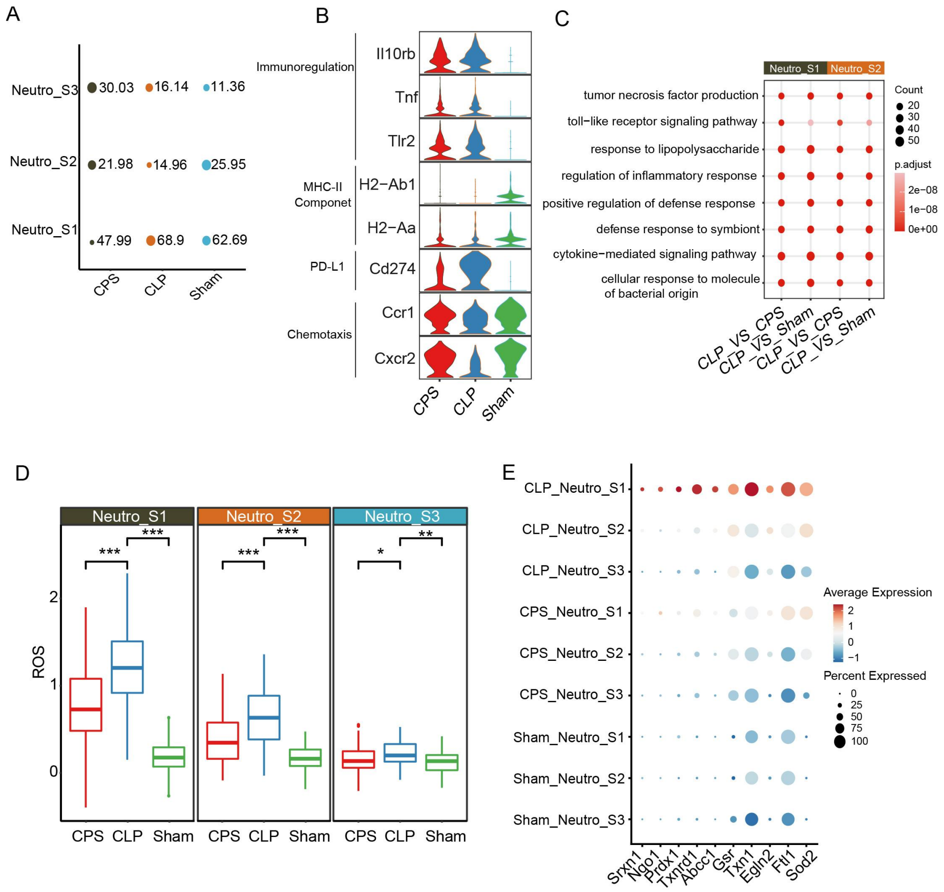


**Figure S6. CPS impacts the immune function and reactive oxygen species production in neutrophils.** (**A**) The dot plot shows the cellular proportion among three neutrophils (sub)types in each group, colored according to (sub)types. (**B**) The violin plot shows the relative expression levels of representative genes of neutrophils of each group in scRNA-seq datasets. (**C**) The bar plot depicts the pathway enrichment analysis of differential genes overlapping up-regulated of neutrophils (sub)types in CLP group compared with Sham group. (**D**) The violin plot shows ROS modules scores in the Sham, the CLP, and the CPS group across Neutro_S1, Neutro_S2 and Neutro_S3. (**E**) The dot plot depicts representative genes of ROS modules in three groups across three neutrophils (sub)types.
